# The need for improved methodology in protein corona analysis

**DOI:** 10.1038/s41467-021-27643-4

**Published:** 2022-01-10

**Authors:** Morteza Mahmoudi

**Affiliations:** Department of Radiology and Precision Health Program, Michigan State University, East Lansing, MI US

**Keywords:** Proteomics, Nanobiotechnology

## Abstract

The protein corona is a key component controlling biological activity, that develops on foreign materials when introduced to biological environments. This comment discusses the risk of errors from poor methodology that can lead to misinterpretation and poor outcomes.

## Abstract (web summary)

The protein corona is a key component controlling biological activity, that develops on foreign materials when introduced to biological environments. This comment discusses the risk of errors from poor methodology that can lead to misinterpretation and poor corona analysis which can impact diagnostic and clinical applications.

The protein corona is a biomolecular shell that forms on the surface of nanoparticles (NPs) during their interactions with biological fluids, which changes over time^1,2^. Aside from the robust characterization of NPs^3–5^, improving the accuracy and robustness of methodologies for preparing the protein corona formed on NPs can significantly improve reproducibility and transparency in nanomedicine, while minimizing misinterpretations. In turn, the methodologies used should be dependent on the intended use. Herein I focus on the role of preparation methodologies on the accuracy and interpretation of biomolecular/protein corona.

## Common methodologies for preparation of protein corona

**Fig. 1:**
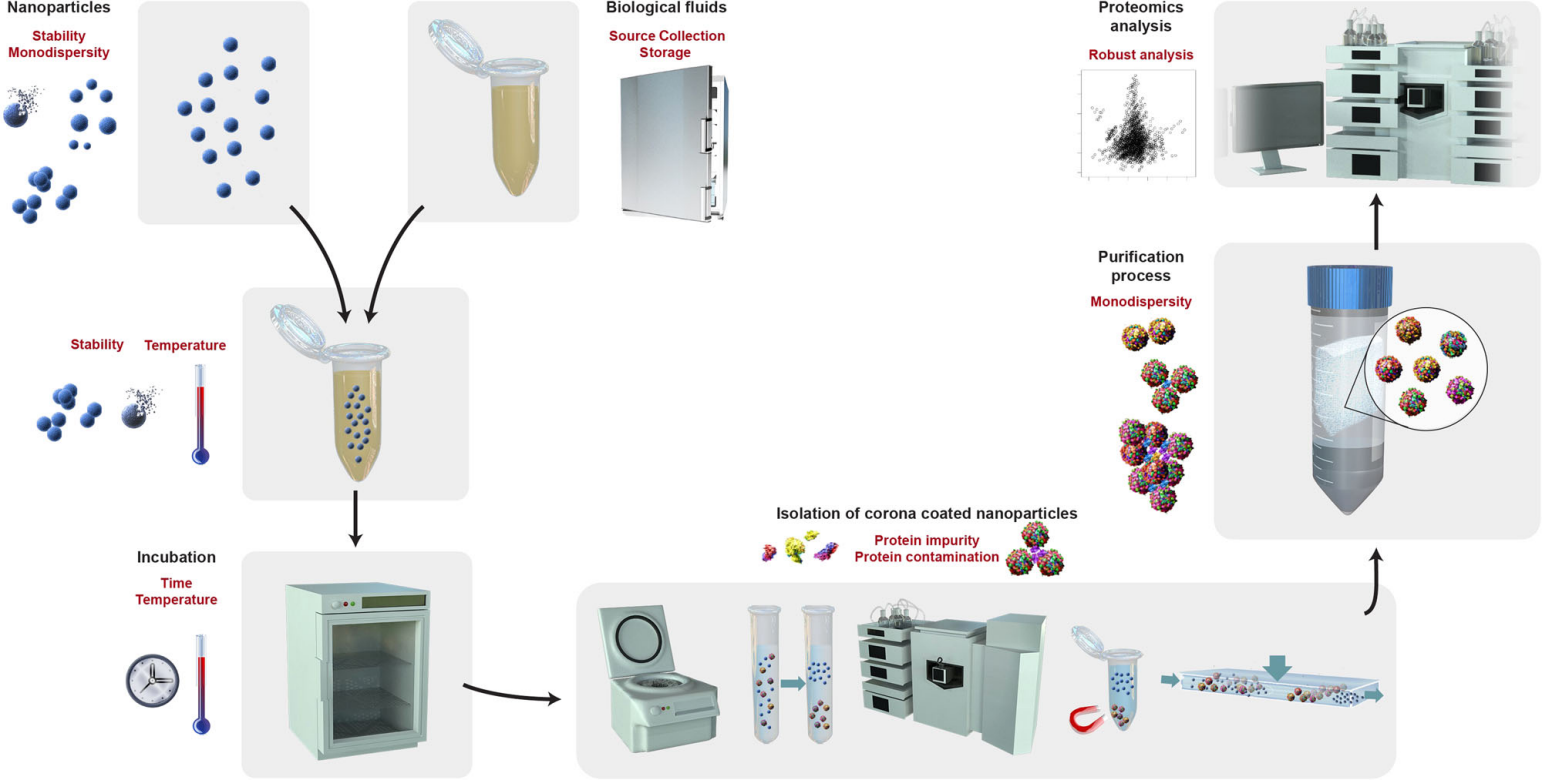
General process for preparation of protein corona and common sources of errors and misinterpretation. General steps for preparation of protein corona in vitro or ex vivo: preparation of nanoparticles and biological fluids; mixing of nanoparticle and biological fluids; incubating the mixture for a certain time and at a specific temperature; isolation of protein corona–coated nanoparticles using the five most common approaches (i.e., centrifugation-based, gradient centrifugation, size exclusion chromatography, magnetic separation, and field-flow fractionation); purification process; and protein corona characterization by proteomic techniques. Red text shows the common methodological features that may cause errors in the outcomes of proteomics analysis of the protein corona (e.g., considering protein impurities in the proteomics data).

The general process for the preparation of the protein corona is as follows: collection and preparation of NPs; collection of biological fluids; mixing NPs and biological fluids; incubation for a certain time at a specified temperature; isolation of protein corona–coated NPs; purification to remove loosely attached and excess proteins; and characterization of protein corona by proteomics approaches (Fig. 1). There are five main methods for isolation of protein corona–coated NPs: centrifugation-based, gradient centrifugation, size exclusion chromatography, magnetic separation, and field flow fractionation. Among these, centrifugation is the most widely used for the collection of corona-coated NPs.

## Common sources of methodological issues (e.g., contamination) in protein corona

Inadequate information about the methods used for the collection and storage of biological fluids (e.g., serum or plasma) can be the first source of errors in protein corona data (Fig. 1). This is mainly because collection and storage methods affect the integrity of proteins and other biomolecules within biological fluids. There are many factors involved in collection and storage all of which can change the biofluid composition. For example, the choice of anticoagulant factors used with blood products can change the biomolecular contents of plasma, thus altering the protein corona composition^6^. As another example, long-term (e.g., multiyear) storage of biological fluids can significantly affect the abundance of many proteins, metabolomes, and lipids which will change the composition of the protein corona^7^. Therefore rigorous quality control and reporting of biological fluids are essential for protein corona analysis.

It is known that incubation temperature can significantly affect the interaction sites of proteins with the surface of NPs, thereby changing the composition of the corona^8^. As such it is advisable that NPs and biofluids should be brought to the desired temperature (usually 37 °C, to mimic human body temperature) before mixing to avoid affecting the proteomics outcomes.

Preparation methods can significantly affect the accuracy of corona analysis and interpretations for both diagnostic and therapeutic purposes. As such methodologies should minimize the introduction of protein impurities/contamination. Failing to consider possible impurities and contamination can cause errors in proteomics outcomes and false-positive and/or false-negative results, harming the targeting/therapeutic efficacy or diagnostic capacity of the protein corona. For example, recent findings revealed that size-exclusion chromatography for the collection of corona-coated NPs is prone to protein contamination due to co-elution of unbound proteins^9^.

Another source of protein contamination comes from corona impurity. Using a combination of imaging and simulation it has recently been reported the protein corona layer may contain a significant amount of small, agglomerated impurities (~<10 nm) unassociated with the corona composition^10^. These impurities could induce significant errors in the outcomes of proteomics analysis, including various types of mass spectroscopy. It was also reported NP concentration plays a crucial role in the creation of such impurities in the corona composition suggesting lower NP concentrations might give more accurate results. It is important, however, to emphasize that every particle in nano-population could be different from others, both due to the unique synthesis condition (e.g., nonequilibrium nucleation and growth reactions) and as a result of further experimentation/interactions. For example, our protein corona analysis of hundreds of monodisperse polystyrene NPs, at single NP level, revealed the existence of a random distribution and concentration of biomolecules in the corona layer^10^. The good news is that the NPs populations may conceal many internal variations when averages (e.g., protein corona profile) are determined.

Conducting the entire protein corona preparation and proteomic analysis in the same vial/wells (mainly for automation purposes) introduces another source of protein contamination due to the well-known phenomenon of protein attachment to well plates^11,12^. Although the use of low-attachment vials/wells may reduce the amount of protein contamination, it cannot eliminate it completely.

The complexity of proteomic instrumentation and the analysis methods for liquid chromatography-mass spectrometry, which introduce many possible sources of variability, are another source of conflicts in protein corona outcomes^13,14^. For example, repeatability and reproducibility in peptide and protein identifications of interlaboratory data set of 144 liquid chromatography-mass spectrometry instruments [i.e., four Thermo linear trap quadrupole (LTQ) and four Orbitrap] revealed the critical role of the employed instrument and data analysis in proteomics outcomes^14^. More specifically, it was found that peptides repeatability in technical replicates were 35−60% depending on the employed instrument (e.g., Orbitrap instruments showed higher repeatability, reproducibility, and stability both for peptides and proteins compared to Thermo LTQ). The repeatable peptides were mostly created from specific proteins (i.e., that creates more distinct peptides) and/or from tryptic cleavage sites^14^. Therefore, failure to consider the role of instrumentation and robust data analysis in experimental replicates of protein corona studies can increase the risk of considering noises as a solid data.

## Common sources of misinterpretation in protein corona

Aside from the above-mentioned technological issues, there are common factors that increase heterogeneity in protein corona data and cause misinterpretation of proteomics outcomes. For example, the use of stable but polydisperse NPs for protein corona preparation can lead to misinterpretation in proteomics outcomes. The main reason is that variations in NP size can significantly affect the composition of the protein corona^15^. To avoid such misinterpretation, NPs used for protein corona preparation should have a low polydispersity index (PDI); e.g., values of ≤0.2 and ≤0.3 are considered as the acceptable homogenous population for polymeric and lipid-based nanocarriers (respectively)^16^.

Careful characterization of the size and polydispersity of NPs used in preparing protein corona (especially regarding mixture and purification steps) is a key means of reducing the possibility of misinterpretation in proteomics outcomes. For example, a fully stable and monodisperse NP may become unstable after being added to the biological fluid. In addition, after collection and purification of the protein corona, if the availability of suitable techniques allows, characterization of the size and polydispersity of the NPs and comparison to the NP’s original characteristics (i.e., prior to mixture with biological fluids) is helpful to identifying possible errors in proteomics data.

Another important source of misinterpretation in corona data comes is variation in the biological fluids used (e.g., bovine-based serum, animal serum/plasma, and human serum/plasma, animal plasma, and human plasma). It is well understood that even subtle changes in the type and composition of biological fluids can significantly change the protein composition of the corona^17^, which is actually the basis of disease identification^18^. Therefore, detailed information on the biological fluid used should be reported and considered in the characterization of protein corona and the proteomics outcomes whenever justifying and/or predicting the safety and diagnostic/therapeutic efficacy of NPs.

## Reducing methodological errors in protein corona

Avoiding the above-described common sources of methodological errors in the preparation of protein corona–coated NPs can significantly improve reproducibility and transparency, facilitating future meta-analysis of protein corona results. To this end, the scientific community (e.g., researchers, editors, and reviewers) should pay more attention to the accuracy of the reported methodologies and characterizations of the various steps of the preparation of protein corona to ensure the validity of proteomics outcomes. It is noteworthy that the validity and accuracy of proteomics analysis of the corona is of crucial importance for successful clinical translation of nanomedicine products for both diagnostic and therapeutic applications. Below are some recommendations intended to minimize methodological errors in various steps of protein corona preparation.

Proper characterization techniques (e.g., dynamic light scattering and/or differential centrifugal sedimentation), according to their standard protocols [e.g., International Organization for Standardization (ISO) 22412:2017 for dynamic light scattering], should be used on NPs in solution before, during, and after the formation of the protein corona to confirm their consistent stability and monodispersity throughout the experiment. Comparing the size and distribution of NPs before and after the formation of the protein corona is a straightforward approach to minimize the possibility of protein contamination through the formation of large aggregates. It is noteworthy that from a physical standpoint, nanoscale objects “experience” water-based solutions as highly viscous fluids (e.g., molasses)^19^, which facilitates protein entrapment between NPs. Without proper and accurate characterization of the NPs’ size and polydispersity during the preparation of the protein corona, the entrapped proteins will be read as data in the proteomics analysis.

Biological fluids should undergo rigorous authentication; the source (e.g., animal or human), type (serum or plasma), and demographic and health information of the donor(s) together with the methodological details of the fluids’ collection and storage should be reported in publications and considered when interpretating protein corona outcomes. It is noteworthy that if pooled plasma/serum are used, the pool size (number of mixed plasmas), health status, sex, and the average age of the pooled plasma should be mentioned in reports.

Proper control samples are essential to rule out the possibility of protein contamination during the collection and purification of protein corona coated NPs. For example, for size exclusion chromatography, the use of biological fluids alone (without NPs) through the entire process is critical. The same control (i.e., biological fluids without NPs) should be used for automated approaches (which likely conduct all steps of the corona preparation in a same vial/well) to determine the degree of protein corona contamination that comes from the interactions of proteins with vials/wells.

The use of concentrated NPs (>0.5 mg/ml) for the preparation of protein coronas may increase the likelihood that protein impurities will form inside the corona shell^10^. These kinds of impurities are hard to detect using conventional approaches and new advanced techniques suitable for the NPs being utilized should be used were possible. Potential impurities need to be accounted for with controls and replication if high concentration of NPs (>0.5 mg/ml) are used. For example, a complementary control, when using concentrated NPs, could be to run the same experiment with a lower concentration of NPs (e.g., in the range of 0.1 mg/ml) but maintain the same NP-to-biological-fluids ratio, and compare the proteomics outcomes.

In summary, robust and accurate methodological approaches, together with vigorous characterization techniques during the preparation of the protein corona, can significantly improve reproducibility and transparency in nanomedicine reports and thereby accelerate the successful clinical translation of nanomedicine technologies. Finally, I would like to emphasize that effective addressing of the reproducibility issues in protein corona and nanomedicine (in general) requires the integrated functioning of all stakeholders. For example, funding agencies can establish specific funding opportunities for deeper investigation on the issue of reproducibility and misinterpretation in nanomedicine research rather than demanding more novelty in the field. The lack of integrated functioning between stakeholders to fully consider the alarming signals of reproducibility in nanomedicine^4,20^ may set the stage for more complexity and uncertainty regarding the timely and effective future success of nanomedicine technologies.
